# Integration of a multi-step heterologous pathway in *Saccharomyces cerevisiae* for the production of abscisic acid

**DOI:** 10.1186/s12934-019-1257-z

**Published:** 2019-11-25

**Authors:** Maximilian Otto, Paulo Gonçalves Teixeira, Maria Isabel Vizcaino, Florian David, Verena Siewers

**Affiliations:** 1 Novo Nordisk Foundation Center for Biosustainability, Department of Biology and Biological Engineering, Chalmers University of Technology, Gothenburg, Sweden; 2 Chalmers Mass Spectrometry Infrastructure, Chalmers University of Technology, Gothenburg, Sweden

**Keywords:** Abscisic acid, Metabolic engineering, Synthetic biology, Sesquiterpenoids, Terpenoids, *Saccharomyces cerevisiae*, *Botrytis cinerea*, Plant hormone

## Abstract

**Background:**

The sesquiterpenoid abscisic acid (ABA) is mostly known for regulating developmental processes and abiotic stress responses in higher plants. Recent studies show that ABA also exhibits a variety of pharmacological activities. Affordable and sustainable production will be required to utilize the compound in agriculture and as a potential pharmaceutical. *Saccharomyces cerevisiae* is an established workhorse for the biotechnological production of chemicals. In this study, we constructed and characterised an ABA-producing *S. cerevisiae* strain using the ABA biosynthetic pathway from *Botrytis cinerea*.

**Results:**

Expression of the *B. cinerea* genes *bcaba1*, *bcaba2*, *bcaba3* and *bcaba4* was sufficient to establish ABA production in the heterologous host. We characterised the ABA-producing strain further by monitoring ABA production over time and, since the pathway contains two cytochrome P450 enzymes, by investigating the effects of overexpressing the native *S. cerevisiae* or the *B. cinerea* cytochrome P450 reductase. Both, overexpression of the native or heterologous cytochrome P450 reductase, led to increased ABA titres. We were able to show that ABA production was not affected by precursor or NADPH supply, which suggested that the heterologous enzymes were limiting the flux towards the product. The *B. cinerea* cytochrome P450 monooxygenases BcABA1 and BcABA2 were identified as pathway bottlenecks and balancing the expression levels of the pathway enzymes resulted in 4.1-fold increased ABA titres while reducing by-product formation.

**Conclusion:**

This work represents the first step towards a heterologous ABA cell factory for the commercially relevant sesquiterpenoid.

## Background

Since its discovery in 1963 [1] the signalling of abscisic acid (ABA) in plants has been studied extensively. As one of the major plant hormones, ABA fulfils a pivotal role in regulating processes like seed dormancy, germination and abiotic stress resistance [2–4]. Its exogenous application on plants was shown to elevate cold [5, 6], salt [7, 8] and drought stress [9, 10]. However, plants are not the only organisms producing and utilising ABA. For example, ABA production was confirmed in phytopathogenic fungi like *Botrytis cinerea* [11], cyanobacteria [12], the animal parasite *Toxoplasma gondii* [13] and mammals including humans [14].

**Fig. 1:**
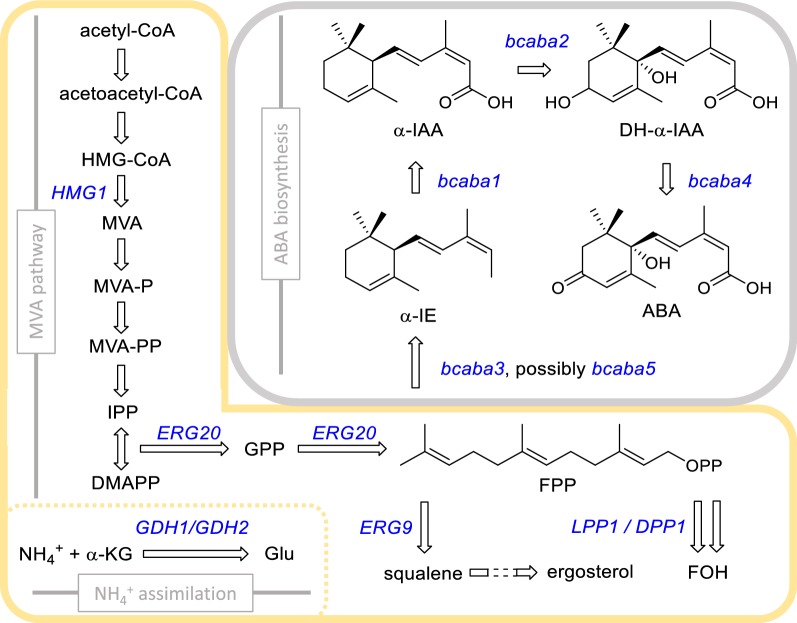
Schematic of relevant metabolic pathways in *S. cerevisiae* and *B. cinerea*. The enzymatic reactions framed in yellow are present in *S. cerevisiae*, while the grey box represents the heterologous reactions catalysed by enzymes from *B. cinerea* (Pathway according to Takino et al. [18]). Genes modified or integrated in at least one of the engineered *S. cerevisiae* strains are shown in blue. *CoA* coenzyme A, *HMG-CoA* 3-hydroxy-3-methylglutaryl-CoA, *MVA* mevalonic acid, *MVA-P* phosphomevalonate, *MVA-PP* diphosphomevalonate, *IPP* isopentenyl diphosphate, *DMAPP* dimethylallyl diphosphate, *GPP* geranyl diphosphate, *FPP* farnesyl diphosphate, *FOH* farnesol, *ABA* abscisic acid, *α-KG* α-ketoglutarate, *Glu* glutamate, *α-IE* α-ionylideneethane, *α-IAA* α-ionylideneacetic acid, *DH-α-IAA* 1′,4′-trans-dihydroxy-α-ionylideneacetic acid

Even though the signalling properties of ABA seem to be conserved between the kingdoms of life, the biosynthetic pathway differs between organisms. In plants, ABA is produced by degradation of C_40_ carotenoids, which are produced in plastids via the 2-C-methyl-d-erythritol 4-phosphate (MEP) pathway [15]. In contrast, *B. cinerea* utilises a pathway in which the sesquiterpenoid is produced directly from the C_15_ molecule farnesyl pyrophosphate (FPP) (Fig. 1) [16, 17]. In the postulated pathway, cyclisation of FPP leads to formation of the ABA core scaffold, which undergoes oxidation by multiple enzymes [17, 18]. In 2006, a gene cluster was described in *B. cinerea* consisting of four genes, including two cytochrome P450 monooxygenase (CYP) genes (*bcaba1* and *bcaba2*), a short-chain dehydrogenase/reductase gene (*bcaba4*), and a gene whose protein sequence did not show any known motifs or homology to characterized proteins (*bcaba3*) [19]. Knock-out experiments confirmed that *bcaba123* are essential for ABA production in *B. cinerea*, whereas *bcaba4* is involved in the pathway but not essential [19, 20]. It was hypothesized that the cyclisation of FPP requires a sesquiterpene cyclase (STC); however, none of the proteins in the gene cluster showed known STC motifs [19, 21]. A study of Izquierdo‐Bueno et al. [21] identified a STC gene named *bcaba5*, which is co-expressed with, but not located in the gene cluster. *Bcaba5* knock-out mutants did not produce ABA [21]. However, Takino et al. [22] evaluated the function of BcABA3 in vitro and showed that the enzyme can catalyse the cyclisation of FPP via an until then unknown reaction mechanism. Furthermore, they showed that ABA could be produced heterologously in an *Aspergillus oryzae* strain expressing *bcaba1234* [22]. The contradicting results of these two studies raise the question if BcABA3 and BcABA5 catalyse the same reaction. Adding to the complexity of the described ABA pathway, Izquierdo‐Bueno et al. [21] identified another gene next to *bcaba5*, in this study called *bcceP450*, which was co-expressed during ABA production and which might thus also be involved in the pathway.

The importance of understanding the biosynthesis of ABA lies in its commercial interest due to the broad spectrum of applications. For example, ABA is already available as the active ingredient of a growth regulator for corn and to control pigmentation of grapes [23, 24]. With the changing climate due to global warming, crop plants will certainly be exposed to more severe abiotic stresses, which is likely to increase the demand for stress regulating compounds like ABA. Recent reviews address possible applications of ABA as a nutraceutical or pharmaceutical [25–27] and several patents have been filed in this regard (see [28, 29] for examples). Effects of ABA include stimulating insulin secretion [30, 31] and anti-inflammatory responses [32] as well as reducing the severity of malaria disease [33].

To exploit the full potential of ABA, cheap production of the compound is necessary, but its chemical synthesis is challenging due to the chiral centre at C-1′ and the double bonds. (*S*)-(+)-ABA with a 2-cis,4-trans configuration is the isomer naturally occurring in plants and *B. cinerea*. Though *B. cinerea* is used for biotechnological production of ABA [34], the limited number of genetic tools available for the native producer makes rational engineering of ABA overproducing strains difficult. Further limitations for the use of the fungus include filamentous growth and an overall slower growth rate compared to other biotechnological workhorses. The yeast *Saccharomyces cerevisiae* is widely used to produce pharmaceuticals and bulk as well as fine chemicals [35, 36]. The organism’s metabolism has been studied in detail and sophisticated genetic tools are available [37–40], which makes it a promising candidate as an ABA cell factory. *S. cerevisiae* is well suited for the expression of fungal CYPs [41] and numerous studies demonstrated its capabilities to overproduce terpenes and terpenoids [42–46].

In this proof-of-concept study, we established a multi-step metabolic pathway in the yeast *S. cerevisiae* to produce ABA. By thoroughly characterising our strain we improved ABA titres 4.1-fold, reduced by-product formation and identified targets for further strain engineering.

## Results

### Establishment of ABA production in three different *S. cerevisiae* strains

To investigate if ABA can be produced in *S. cerevisiae* with the *B. cinerea* genes described in the literature thus far, we first integrated all heterologous genes in the genome that were reported to be (potentially) involved in the pathway: *bcaba1*, *bcaba2*, *bcaba3*, *bcaba4*, *bcaba5* and *bcceP450* [19, 21]. In *B. cinerea*, *bcaba1*, *bcaba2*, *bcaba3* and *bcaba4* are located in the ABA gene cluster [19], while *bcaba5* and *bcceP450* are colocalized on a different chromosome [21]. Since *bcaba1*, *bcaba2* and *bcceP450* encode CYPs and the native *S. cerevisiae* NADPH cytochrome P450 reductase (CPR) might not be compatible with these heterologous proteins, we also integrated the corresponding *B. cinerea* gene, referred to as *bccpr1*.

Precursor and co-factor supply are pivotal for cell factories and can directly affect production. To determine the effect of varying precursor and co-factor supply in ABA production, three different background strains were analysed: (i) CEN.PK113-5D wild-type (named 5D), (ii) CEN.PK113-5D overexpressing the truncated form of *HMG1* (named 5D-tHMG1) [47], and (iii) the strain SCIGS22a, a platform strain for sesquiterpene production, which was previously used for the efficient production of the apocarotenoid β-ionone [43]. All postulated ABA pathway genes were integrated into the genome and the strains were named: (i) DABA1, (ii) TABA1 and (iii) SABA1, respectively (see Table 1; Fig. 7 in “Methods” section). Both background strains 5D-tHMG1 and SCIGS22a have increased flux through the MVA pathway by continuously overexpressing the truncated version of 3-hydroxy-3-methylglutaryl-CoA reductase (*tHMG1*), coding for a highly regulated enzyme in this pathway [47–49]. In SCIGS22a (constructed by Scalcinati et al. [50] and López et al. [43], see Fig. 1 for pathway schematic) FPP supply is further increased by overexpressing the FPP synthase gene *ERG20* and deletion of *LPP1* and *DPP1*, both encoding enzymes catalysing the dephosphorylation of FPP [51–53]. The native promoter of *ERG9* was exchanged for the glucose-dependant promoter P_*HXT1*_ [53, 54], thereby avoiding drainage of FPP towards ergosterol biosynthesis during growth in low-glucose conditions. Lastly, by removing a NADPH-dependant glutamate dehydrogenase reaction of ammonium assimilation (deletion of *GDH1*) and promoting a NADH-dependant glutamate dehydrogenase reaction (overexpression of *GDH2*), the NADH-NADPH balance is shifted in favour of NADPH [50, 55, 56]. NADPH is required by Hmg1 for converting HMG-CoA to MVA and for the oxidation of the ABA backbone by the *B. cinerea* CYPs.

**Table 1:** *S. cerevisiae* strains used in this study

	Strain name	Genotype	References
Background strains	CEN.PK113-5D	*MAT***a** *MAL2*-*8* ^*c*^ *SUC2 ura3*-*52*	[76], provided by P. Kötter, University of Frankfurt, Germany
	5D-tHMG1	*MAT***a** *MAL2*-*8* ^*c*^ *SUC2 ura3*-*52* P_*TEF1*_ -*tHMG1*	This study
	SCIGS22a	*MAT***a** *MAL2*-*8* ^*c*^ *SUC2 ura3*-*52 lpp1*Δ::*loxP dpp1*Δ::*loxP* P_*ERG9*_ Δ::*loxP*-P_*HXT1*_ *gdh1*Δ::*loxP* P_*TEF1*_ -*ERG20* P_*PGK1*_ -*GDH2* P_*TEF1*_ -*tHMG1*	[43]
Strains with *B. cinerea* genes	DABA1	*MAT***a** *MAL2*-*8* ^*c*^ *SUC2 ura3*-*52* P_*PGK1*_-*bcaba1* P_*PGK1*_-*bcaba2* P_*TEF1*_-*bcaba3* P_*TEF1*_-*bcaba4* P_*PGK1*_-*bcaba5* P_*TEF1*_-*bccpr1* P_*PGK1*_-*bcceP450*	This study
	TABA1	*MAT***a** *MAL2*-*8*^*c*^ *SUC2 ura3*-*52* P_*TEF1*_ -*tHMG1* P_*PGK1*_-*bcaba1* P_*PGK1*_-*bcaba2* P_*TEF1*_-*bcaba3* P_*TEF1*_-*bcaba4* P_*PGK1*_-*bcaba5* P_*TEF1*_-*bccpr1* P_*PGK1*_-*bcceP450*	This study
	SABA1	*MAT***a** *MAL2*-*8* ^*c*^ *SUC2 ura3*-*52 lpp1*Δ::*loxP dpp1*Δ::*loxP* P_*ERG9*_ Δ::*loxP*-P_*HXT1*_ *gdh1*Δ::*loxP* P _*TEF1*_ -*ERG20* P_*PGK1*_ -*GDH2* P_*TEF1*_-*tHMG1* P_*PGK1*_-*bcaba1* P_*PGK1*_-*bcaba2* P_*TEF1*_-*bcaba3* P_*TEF1*_-*bcaba4* P_*PGK1*_-*bcaba5* P_*TEF1*_-*bccpr1* P_*PGK1*_-*bcceP450*	This study
	TABA2	*MAT***a** *MAL2*-*8*^*c*^ *SUC2 ura3*-*52* P_*TEF1*_ -*tHMG1* P_*PGK1*_-*bcaba1* P_*PGK1*_-*bcaba2* P_*TEF1*_-*bcaba3* P_*TEF1*_-*bcaba4* P_*PGK1*_-*bcaba5* P_*TEF1*_-*bccpr1*	This study
	TABA3	*MAT***a** *MAL2*-*8* ^*c*^ *SUC2 ura3*-*52* P_*TEF1*_ -*tHMG1* P_*PGK1*_-*bcaba1* P_*PGK1*_-*bcaba2* P_*TEF1*_-*bcaba3* P_*TEF1*_-*bcaba4* P_*TEF1*_-*bccpr1*	This study
	SABA3	*MAT***a** *MAL2*-*8* ^*c*^ *SUC2 ura3*-*52 lpp1*Δ::*loxP dpp1*Δ::*loxP* P_*ERG9*_ Δ::*loxP*-P_*HXT1*_ *gdh1*Δ::*loxP* P _*TEF1*_ -*ERG20* P_*PGK1*_ -*GDH2* P_*TEF1*_-*tHMG1* P_*PGK1*_-*bcaba1* P_*PGK1*_-*bcaba2* P_*TEF1*_-*bcaba3* P_*TEF1*_-*bcaba4* P_*TEF1*_-*bccpr1*	This study
	TABA4	*MAT***a** *MAL2*-*8* ^*c*^ *SUC2ura3*-*52* P_*TEF1*_ -*tHMG1* P_*PGK1*_-*bcaba1* P_*PGK1*_-*bcaba2* P_*TEF1*_-*bcaba4* P_*PGK1*_-*bcaba5* P_*TEF1*_-*bccpr1*	This study
	TABA5	*MAT***a** *MAL2*-*8* ^*c*^ *SUC2 ura3*-*52* P_*TEF1*_ -*tHMG1* P_*PGK1*_-*bcaba1* P_*PGK1*_-*bcaba2* P_*TEF1*_-*bcaba3* P_*TEF1*_-*bcaba4* P_*TEF1*_-*NCP1*	This study
	TABA6	*MAT***a** *MAL2*-*8* ^*c*^ *SUC2 ura3*-*52* P_*TEF1*_ -*tHMG1* P_*PGK1*_-*bcaba1* P_*PGK1*_-*bcaba2* P_*TEF1*_-*bcaba3* P_*TEF1*_-*bcaba4*	This study
	S3S0	SABA3 with p416 empty	This study
	S3S1	SABA3 with p416-ABA1	This study
	S3S2	SABA3 with p416-ABA2	This study
	S3S3	SABA3 with p416-ABA3	This study
	S3S4	SABA3 with p416-ABA4	This study
	S3S12	SABA3 with p416-ABA1 + 2	This study

**Fig. 2:**
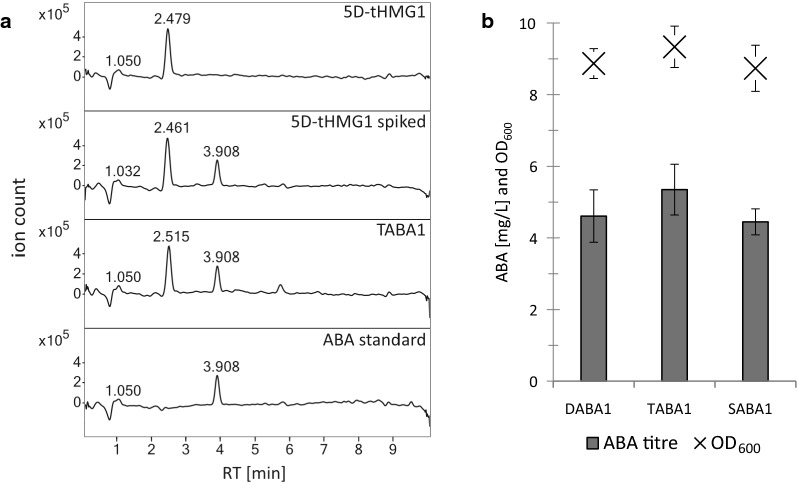
HPLC–MS analysis of ABA-producing strains and controls. **a** Chromatograms of extracted supernatants after 48 h of cultivation of the strains 5D-tHMG1, 5D-tHMG1 spiked with ABA standard, TABA1 (5D-tHMG1 carrying *bcaba12345, bccpr1 and bcceP450*), and (*S*)-(+)-ABA standard dissolved in methanol. One replicate is displayed per strain. Retention time is displayed on top of the peaks. The chromatograms were smoothed and a blank methanol run was subtracted to remove impurities. The strains DABA1 and SABA1 showed the same chromatogram peaks as TABA1 (Figure S1 in Additional file 1). Chromatograms of all strains without the subtraction of the methanol blank run can be found in Figure S1 in Additional file 1. *RT* retention time. **b** ABA titre in supernatant and OD_600_ of strains containing *bcaba12345*, *bccpr1* and *bcceP450*. Strains were cultivated for 48 h in minimal medium supplemented with uracil. DABA1 is based on the genetic background of 5D, TABA1 is based on 5D-tHMG1 and SABA1 is based on SCIGS22a. Average ABA titres and maximum OD_600_ were calculated from three independent biological replicates

After 48 h of cultivation in minimal medium supplemented with uracil, supernatant and the lyophilised cell pellet of the background strains and the engineered strains were extracted with ethyl acetate containing 0.5% formic acid (supernatant) or methanol (pellet). The (*S*)-(+)-ABA standard showed a peak at 3.900 min ± 0.050 (Fig. 2a). This peak was visible for all strains containing the *B. cinerea* genes, as well as for the control samples spiked with ABA standard, whereas the peak was not present in any of the control strains 5D, 5D-tHMG1 and SCIGS22a (Fig. 2a, Figure S1 in Additional file 1). By comparing the MS spectra of the ABA standard to the engineered strains, the peak at 3.9 min was unambiguously identified as ABA (Figure S2 in Additional file 1). The strain TABA1 showed the highest average concentration of ABA with 4.7 mg/L, followed by DABA1 and SABA1, both producing ca. 4 mg/L (Fig. 2b).

With this experiment, ABA production was confirmed for the three strains expressing *bcaba12345*, *bccpr1* and *bcceP450*. However, no strain produced significantly higher amounts than another. ABA was not detected by HPLC–MS in the freeze-dried cell pellets (20 mg), neither by analysing the total-ion chromatogram nor by checking for an ion with the expected *m/z* of 265 in the extracted-ion chromatogram (data not shown). The samples spiked with ABA standard were used to estimate the efficiency of the extraction process. It was found that with the supernatant extraction method (ethyl acetate with formic acid) 93% of the ABA standard added to the sample before the extraction was retrieved and with the pellet extraction method (methanol) 89% of ABA was retrieved. This confirms that the absence of ABA in the cell pellet is not due to using a different extractant. ABA seems to be either transported or can freely diffuse through the cell membrane. A structurally similar but more hydrophobic sesquiterpenoid, artemisinic acid, was found to be bound to the cell surface and removed by washing with alkaline buffer [57]. Presumably this could also be the case with ABA. However, even when the water washing step was omitted, no ABA was detectable in the cell pellet (data not shown).

No pronounced differences in growth rate or maximum OD_600_ were observed in the growth profiler between the ABA producing strains and the background strains (Fig. 2b, Figure S5 in Additional file 1). After 48 h of cultivation the pH in the supernatant dropped from pH 6.5 before the inoculation to approximately pH 5.6 for all strains.

The FPP overproducing strain SCIGS22a is expected to provide the highest amount of precursors and co-factors [43]. However, the ABA titre of SABA1 was not significantly higher than for DABA1 or TABA1. These results suggest that precursor supply is not limiting ABA production in the strains.

### Effects of *bcaba3*, *bcaba5* and *bcceP450* on ABA production

Recently, two conflicting studies were published about the number of genes necessary for ABA production in *B. cinerea.* Takino et al. showed that the four genes of the gene cluster, *bcaba1234*, are sufficient to produce ABA in *Aspergillus oryzae* [22]. However, the results of Izquierdo‐Bueno et al. demonstrated that *bcaba5*, encoding an STC, is essential for ABA production in *B. cinerea* [21]. *Bcaba5* is not located in the ABA gene cluster, but in *bcaba5* knock-out mutants no ABA could be detected and this phenotype could be rescued by reintroducing the gene [21]. Takino et al. suggested that *bcaba5* might be involved in ABA biosynthesis but is not essential [22]. Furthermore, expression analysis in the native host revealed another gene encoding a CYP (*bcceP450* located upstream of *bcaba5*) that is co-expressed with *bcaba123* and could play a role in ABA biosynthesis [21].

To bring more insight into which genes are essential for ABA biosynthesis and which genes might be beneficial for ABA production in a yeast cell factory, we constructed *S. cerevisiae* strains lacking *bcaba3*, *bcaba5* and/or *bcceP450* (see Table 1; Fig. 7 in “Methods” section). The strains are based on the background strain 5D-tHMG1, which showed the highest ABA titre in the previous experiment. The strains TABA1 (containing *bcaba12345, bccpr1* and *bcceP450*), TABA2 (lacking the *bcceP450*), TABA3 (lacking *bcceP450* and *bcaba5*), and TABA4 (lacking *bcceP450* and *bcaba3*) were compared for ABA production and growth.

**Fig. 3:**
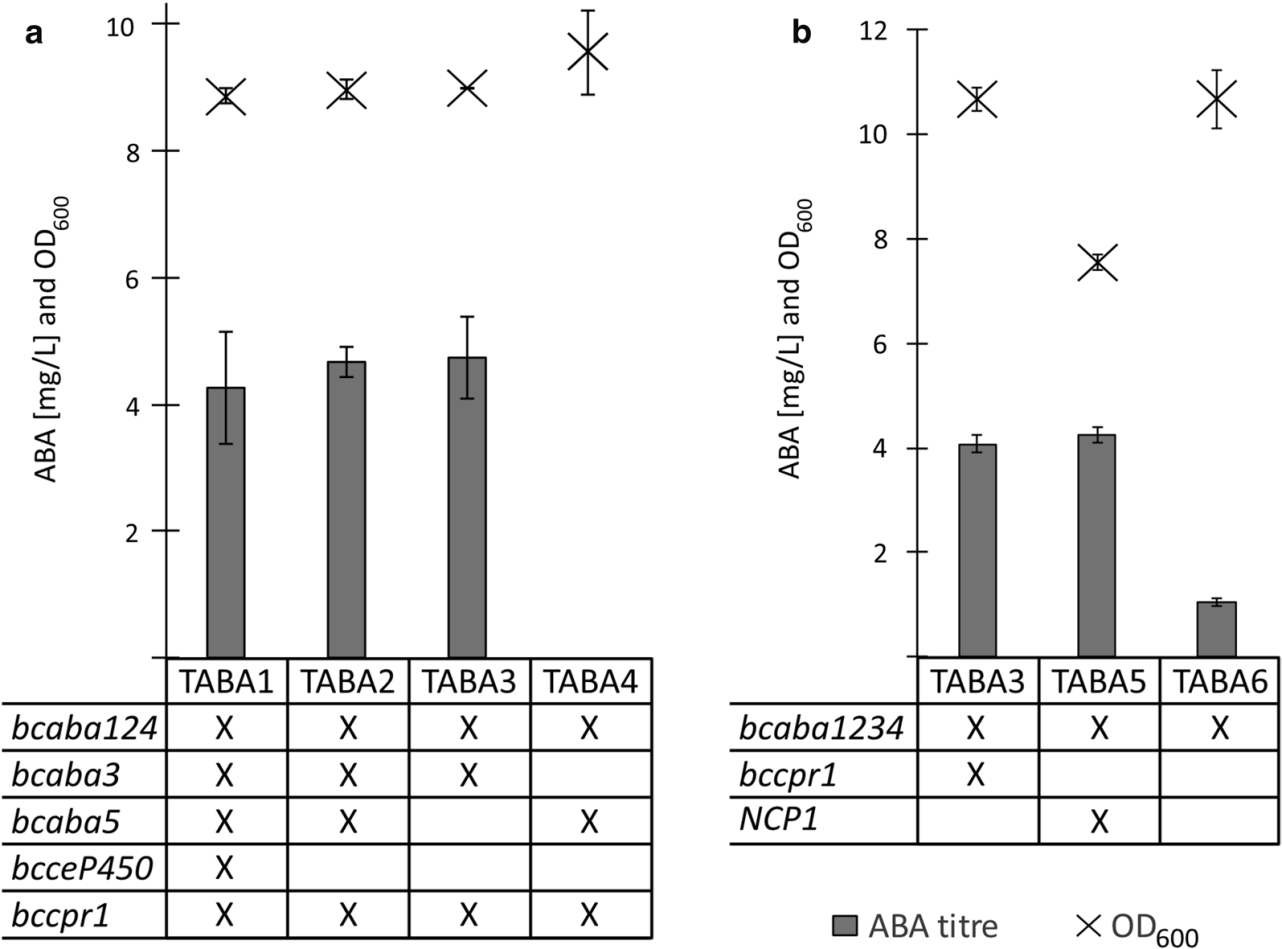
ABA titre in supernatant and OD_600_ of strains based on 5D-tHMG1. Strains were cultivated for 48 h in minimal medium supplemented with uracil. Average ABA titres and maximum OD_600_ were calculated from three independent biological replicates. Tables below the graphs list heterologous genes present in the different strains or in the case of *NCP1* a native overexpressed gene. **a** Effect of *bcaba3, bcaba5 and bcceP450* on ABA titres. No ABA was detected for TABA4. The strains were cultivated in shake flasks. **b** Effect of CPR overexpression on ABA titres. TABA3 expresses the *B. cinerea* CPR gene *bccpr1*, TABA5 overexpresses the native *S. cerevisiae* CPR gene *NCP1* and TABA6 does not overexpress a CPR. The strains were cultivated in deep-well plates

The HPLC–MS analysis showed that TABA2 and TABA3 produce ABA, whereas TABA4 does not (Fig. 3a). Addition of *bcaba5* or *bcceP450* did not have a positive effect on the ABA production. The strains produced between 4.2 and 4.7 mg/L ABA, with no significant difference between them. As in the previous experiment, no ABA was detected in the cell pellets. Compared to TABA3, no additional peaks were observed for TABA1, TABA2 or TABA4, neither in the supernatant nor in the cell pellet (Figure S3 and Figure S4 in Additional file 1). As before, the strains expressing the *B. cinerea* genes showed no differences in growth behaviour compared to the background strain 5D-tHMG1 (Figure S6 in Additional file 1). The pH in the supernatant at the time of harvest was between pH 5.5 and pH 5.7.

CPRs mediate the electron transfer from NADPH to CYPs and are essential for their function. We evaluated the importance of CPR overexpression and investigated if the *S. cerevisiae* CPR Ncp1 is compatible with the *B. cinerea* enzymes. Two new strains were constructed: TABA5 overexpressing *NCP1* and TABA6 in which no CPR is overexpressed. The strain TABA3 expresses the heterologous *bccpr1*. The strains overexpressing a CPR, TABA3 and TABA5, showed 3.5-fold increased ABA titres compared to TABA6 (Fig. 3b). There was no difference in ABA titres between TABA3 and TABA5, even though *NCP1* overexpression in TABA5 resulted in a 30% lower OD_600_ compared to the other two strains.

In summary, our results show that in *S. cerevisiae bcaba5* is not essential for ABA production in the engineered *S. cerevisiae* strain. This is in line with the results of Takino et al. [22]. Integration of *bcaba5* and *bcceP450* showed no positive effects on ABA titres, while overexpression of a CPR significantly affects ABA titres. The *S. cerevisiae* CPR appears to be compatible with the heterologous CYPs, but its overexpression causes a growth defect.

### ABA production over 58 h of cultivation

**Fig. 4:**
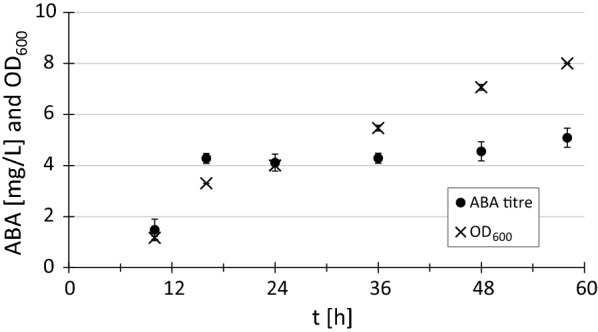
Time series of OD_600_ and ABA titre in supernatant of TABA3. ABA titres were determined via HPLC–MS after 10 h, 16 h, 24 h, 36 h, 48 h and 58 h. Average ABA titres and OD_600_ were calculated from three independent biological replicates

Next, we investigated at which time points of a typical batch culture ABA production occurs. We presumed that further characterisation of the strain could lead to future engineering targets. 200 mL cultures of the strain TABA3 and its background strain 5D-tHMG1 were sampled over a period of 58 h. The OD_600_ was monitored to follow the growth profile and HPLC–MS samples (15 mL) were taken after 10 h, 16 h, 24 h, 36 h, 48 h and 58 h. At 10 h, small amounts of ABA were detectable in the supernatant of all TABA3 replicates by HPLC–MS (Fig. 4). A strong increase in ABA titre in the supernatant is visible after 16 h. Afterwards, the ABA titre in the supernatant remains at approximately 4 mg/L until 48 h. At 58 h the ABA concentration reached 5.1 mg/L, similar to the titre determined in the previous experiment (Fig. 3a). ABA is predominantly produced during growth in glucose, even though strong constitutive promoters (P_*TEF1*_ and P_*PGK1*_) were used to drive the ABA gene expression.

### Screening for accumulation of pathway intermediates

Individual enzymes of multi-step metabolic pathways can have different catalytic activities, which can result in accumulation of pathway intermediates or formation of side-products and limit the flux towards the product of interest. By screening for intermediates, pathway imbalances can be spotted and subsequently optimised. We proposed that high-resolution mass spectrometry (HPLC-HRMS) would provide the necessary sensitivity and accuracy to do so. Extracted supernatant and pellet samples of TABA3 and its background strain 5D-tHMG1 were analysed (samples from previous experiment obtained after 24 h of cultivation, see Fig. 4). To avoid bias, we screened for all compounds with a *m/z* between 200 and 270 (α-ionylideneethane: *m/z* 205 [M+H], ABA: *m/z* 265 [M+H]), instead of screening for known or postulated intermediates. By comparing to molecular features in the background strain, it was possible to distinguish between metabolites unique to the ABA-producing strain and compounds that are part of the native *S. cerevisiae* metabolism. In the supernatant, 76 unique molecular features were detected, whereas in the pellet 17 unique molecular features could be found. Tables with the detected masses can be found in Additional file 2.

**Fig. 5:**
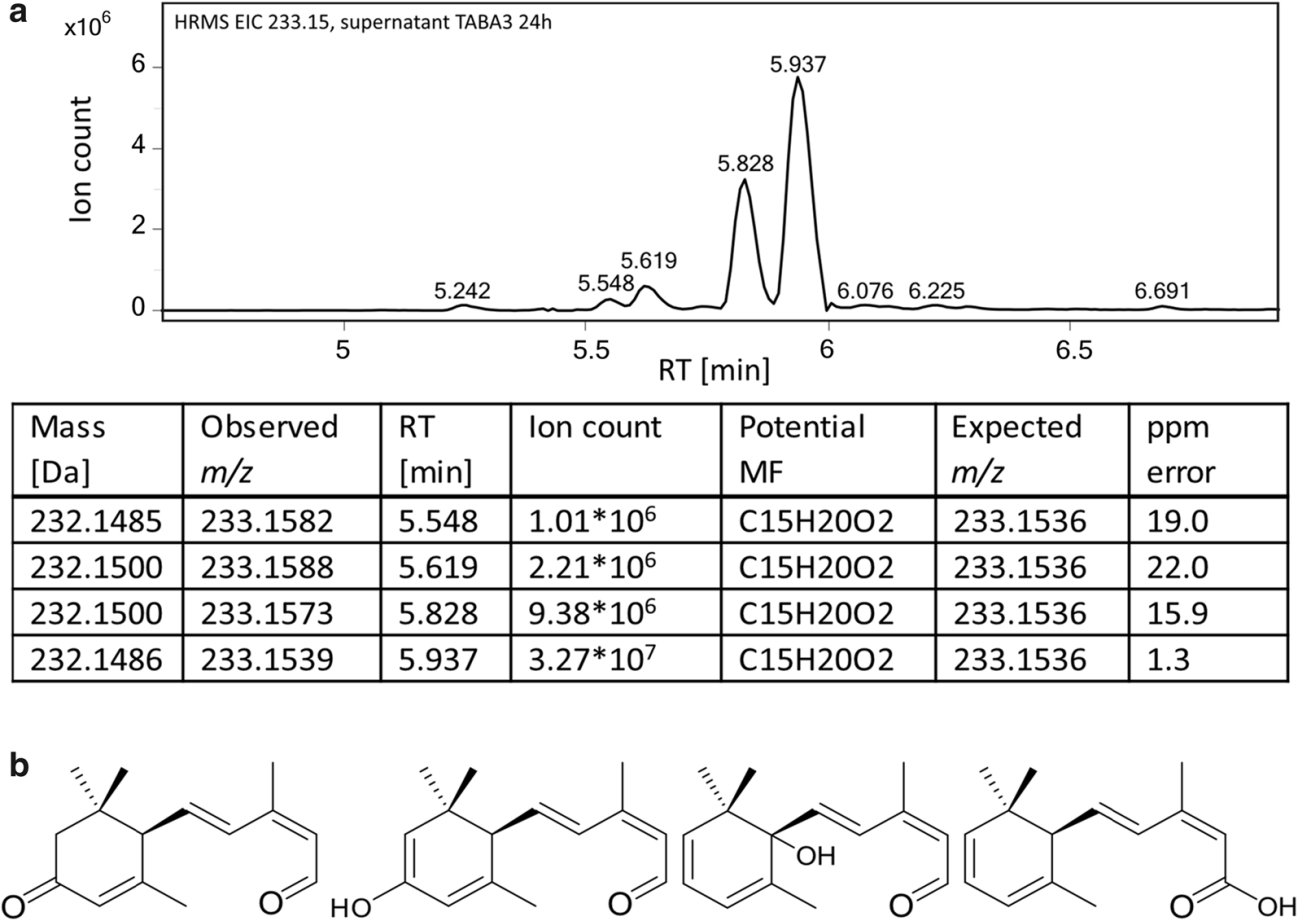
Results of HPLC-HRMS analysis and postulated molecular structures for MZ233. **a** Excerpt of extracted ion chromatogram (EIC) for *m/z* 233.15 from HPLC-HRMS analysis of the supernatant of TABA3 at 24 h. Information about peaks with abundance > 10^6^ is listed in the table. No corresponding peaks were observed in 5D-tHMG1. *RT* retention time, *MF* molecular formula. **b** Possible structures for the compound with *m/z* 233.1539 at 5.9 min (named MZ233). Structures are based on the assumption that the compound originates from the ABA backbone and has the molecular formula C_15_H_20_O_2_

In the supernatant, two compounds were found with ion counts similar to ABA (> 10^7^): *m/z* 233.1539 (retention time 5.9 min, labelled as MZ233) and *m/z* 247.1298 (retention time 4.5 min). The *m/z* 247.1298 was also found in the ABA standard (Figure S2 in Additional file 1) and was identified as a degradation product of ABA formed by loss of H_2_O. The high abundance of *m/z* 233.1539, suggests that it is a compound involved in, or originating from, the ABA pathway. Besides the peak at 5.9 min, several other compounds with similar *m/z* were detected with lower ion-counts and slightly different retention times (at 5.5 min, 5.6 min and 5.8 min, see Fig. 5a and Additional file 1).

The proposed structures of MZ233 (Fig. 5b), with a molecular formula of C_15_H_20_O_2_, is supported by the high-resolution mass (ppm error 1.3 from expected *m/z*, Fig. 5a). However, the other compounds at 5.5, 5.6 and 5.8 min show ppm errors of 19.0, 22.0 and 15.9 respectively, suggesting that these are distinct compounds, possibly with other molecular formulae. The accumulating compound MZ233 appears to be an intermediate or side-product of the ABA pathway, which is missing two oxidation steps. To our knowledge, no *B. cinerea* ABA intermediate with the molecular mass of 232 Da has been reported in the literature.

A *m/z* of 233.156 at 5.9 min was detected in the cell pellet and was the only compound reaching ion counts higher than 10^6^ (Additional file 2). In contrast to the previous HPLC–MS experiments, ABA was detectable in the cell pellet with the increased sensitivity of the HPLC-HRMS. The ion count for ABA was below 10^5^ (Additional file 2).

We detected masses correlating with known or postulated ABA pathway intermediates reported in previous studies [17–19, 58], namely α-ionylideneethane (204.19 Da), α-ionylideneacetic acid (234.16 Da), 1′-deoxy ABA (248.14 Da) and 1ʹ,4ʹ-trans-dihydroxy-α-ionylideneacetic acid (266.15 Da) (Additional file 2). These masses were only detected in TABA3 and not found in 5D-tHMG1. However, for unambiguous identification of the compounds chemical standards would be required.

### Identification of ABA pathway bottlenecks

Seeing that strains with different precursor and co-factor supply did not differ in ABA titres (Fig. 2b) and furthermore that a pathway intermediate or side-product is accumulating (Fig. 5), we assumed that the abundance or activity of at least one of the *B. cinerea* enzymes is limiting ABA production. To investigate this hypothesis, a second copy of *bcaba1*, *bcaba2*, *bcaba3* or *bcaba4* was expressed from a centromeric plasmid in SABA3. The strain SABA3 was chosen since it provides higher amounts of FPP and NADPH [43, 50], which could become limiting once expression levels of the ABA pathway enzymes are optimised.

**Fig. 6:**
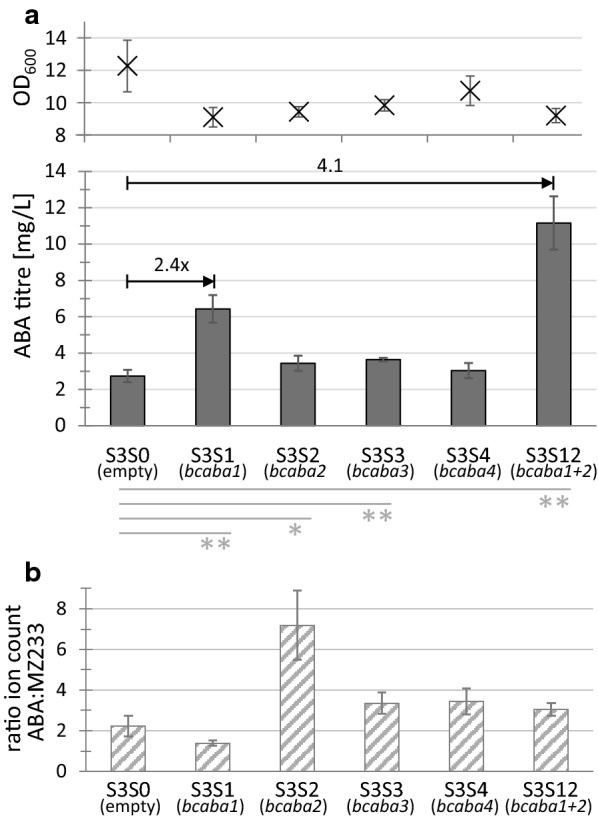
Plasmid-based expression of a second ABA gene in strain SABA3. The gene(s) expressed from the plasmids are listed in brackets below the strains. All genes were expressed from p416 GPD controlled by P_*TDH3*_ or P_*TEF1*_ and P_*TDH3*_ in case of S3S12. The control strain S3S0 carries an empty plasmid. Measurements were taken after 48 h of cultivation in minimal medium. Average and standard deviation were calculated from three independent biological replicates. **a** ABA titre in culture supernatant and OD_600_. Grey lines below the graph show statistical significance compared to the control strain S3S0 (*significant, p < 0.05; **highly significant, p < 0.01). **b** Ratio of ion counts for ABA and MZ233. The ratios of S3S1, S3S3, S3S4 and S3S12 were significantly different compared to the control strain S3S0 (p < 0.05), while S3S2 showed a highly significant difference (p < 0.01)

Expressing an additional copy of *bcaba1* or *bcaba3* resulted in a highly significant (p < 0.01) increase in ABA titres, while *bcaba2* had a significant (p < 0.05) effect compared to the strain carrying the empty vector. ABA concentrations in the supernatant were increased 2.4-fold with an additional copy of *bcaba1* (Fig. 6a, strain S3S1), and 1.3-fold with *bcaba2* or *bcaba3* (Fig. 6a, strains S3S2 and S3S3 respectively). Expression of *bcaba4* from a plasmid (S3S4) did not result in a significant increase in ABA levels.

We further investigated if the additional gene copies affect the amount of MZ233, a presumed ABA intermediate or side-product. The ratio between the ion count of ABA and the ion count of MZ233 was calculated for all plasmid-carrying strains (Fig. 6b). All strains showed significant differences (p < 0.05) in the ABA:MZ233 ratio compared to the empty vector control, with S3S2 showing a highly significant difference (p < 0.01). The control strain S3S0 accumulated about 2.2-fold more ABA than MZ233. For S3S1 the ratio dropped to 1.4, indicating that MZ233 accumulates in this strain; whereas in S3S2, low amounts of MZ233 were produced, resulting in an ABA:MZ233 ratio of 7.2. The ratio for S3S3 and S3S4 are higher than for S3S0, reaching 3.3 and 3.4 respectively.

These results indicate that ABA production is mainly limited by BcABA1 activity. Furthermore, we conclude that MZ233 is an intermediate or side-product in the ABA pathway downstream of BcABA1 but upstream of BcABA2, since we saw a decrease in the ABA:MZ233 ratio for S3S1 and an increase for S3S2 (Fig. 6b). MZ233 accumulation can be avoided with higher BcABA2 activity, which would likely increase the pathway flux towards ABA. Based on these results we constructed the strain S3S12, carrying a plasmid with both genes *bcaba1* and *bcaba2*. S3S12 showed the highest ABA titre of all our strains with a 4.1-fold increase in comparison to S3S0 (Fig. 6a). Compared to S3S0 and S3S1, less MZ233 seems to accumulate with an ABA:MZ233 ratio of 3 (Fig. 6b).

After 48 h, the OD_600_ of strains expressing additional gene copies were slightly lower than for the control strain (Fig. 6a).

In summary, we could show that the activities of the *B. cinerea* CYPs BcABA1 and BcABA2 are bottlenecks for ABA production in our strains and that additional gene copies can increase the flux towards ABA.

## Discussion

In this study, we demonstrate for the first time the heterologous production of the sesquiterpenoid ABA in *S. cerevisiae*, via a four-enzyme metabolic pathway from *B. cinerea*, thereby paving the way to establish a cell factory for a compound relevant for the agricultural, and potentially also, the pharmaceutical industry.

When the *B. cinerea* ABA gene cluster consisting of genes *bcaba1234* was first discovered, the lack of a gene with known STC motifs was surprising [19]. In the biosynthesis of the sesquiterpenoid botrydial in *B. cinerea* an STC catalyses the initial cyclisation of FPP [59, 60]. It was therefore likely that the same class of protein is also involved in the cyclisation of FPP to the ABA backbone α-ionylideneethane. Izquierdo‐Bueno et al. [21] found an STC gene, named *bcaba5*, outside of the original gene cluster in *B. cinerea*, which showed increased expression when ABA was produced. In that study, knock-out of the gene abolished ABA production in *B. cinerea*. The phenotype could be rescued by reintroducing *bcaba5*, seemingly confirming that BcABA5 is a key enzyme in the pathway [21]. However, a more recent study by Takino et al. [22] investigated *bcaba3*, a gene of the original gene cluster encoding an enzyme with hitherto unknown function and no known motifs. Their in vitro assays showed that BcABA3 can convert FPP to α-ionylideneethane. Furthermore, ABA could be detected in an *A. oryzae* strain harbouring *bcaba1234* [22], thereby contradicting the findings of Izquierdo‐Bueno et al. [21]. In this study, we confirmed the findings of Takino et al., the four genes in the *B. cinerea* ABA gene cluster, *bcaba1234*, are sufficient to produce ABA and *bcaba5* is not essential. However, it could still be possible that the co-expression of *bcaba5* enhances ABA production. The yield of the sesquiterpenoid artemisinic acid, a precursor for the anti-malaria drug artemisinin, was increased two-fold by expressing non-essential pathway genes from the native host [46]. *Bcaba5* and the CYP *bcceP450* are expressed during ABA production in *B. cinerea*, indicating that they could be involved in the pathway [21]. Integration of *bcaba5* and *bcceP450* did not increase ABA titres for our strains (Fig. 3a). Nonetheless, further analysis will be necessary to confirm unambiguously that BcABA5 and BcceP450 are not involved in ABA biosynthesis. The proteins might still contribute to ABA production once other limitations are removed. It is also possible that the proteins are not folded properly or that they are otherwise not active in the heterologous host due to, e.g. allosteric regulation.

In the HPLC-HRMS analysis an ion with the *m/z* 233.1538 [M+H], named MZ233, was detected with an abundance similar to ABA (Fig. 5a, Additional file 2). MZ233 was neither detected in the background strains nor in the ABA standard, suggesting that it is an ABA pathway intermediate or side-product. Based on the postulated ABA pathway, we proposed four structures with the molecular formula of C_15_H_20_O_2_ that are supported by the observed high-resolution mass (1.3 ppm error, Fig. 5a). By expressing additional copies of *bcaba1* or *bcaba2* from a plasmid and subsequently observing that the relative amount of MZ233 changed (Fig. 6b), we confirmed that MZ233 is an ABA-related compound. The results show that BcABA1 acts upstream of MZ233 while BcABA2 acts downstream of the compound. To our knowledge no ABA intermediate with the molecular mass of 232 Da [M] has been reported in the literature. Takino et al. [18] investigated the *B. cinerea* ABA pathway by expressing the genes *bcaba1*, *bcaba2*, *bcaba3* and *bcaba4* in *A. oryzae* and found that α-ionylideneethane is oxidised by BcABA1 to form α-ionylideneacetic acid, which is then converted to 1ʹ,4ʹ-trans-dihydroxy-α-ionylideneacetic acid by BcABA2 (Fig. 1). α-Ionylideneacetic acid has a molecular mass of 234 Da. MZ233 is likely to originate from α-ionylideneacetic acid and may only differ in one double bond at the 4′ position (Fig. 5b, structure on the right). Promiscuous activity of an *S. cerevisiae* enzyme could be responsible for the double bond formation, making MZ233 a side-product, which accumulates if limited activity of BcABA2 restricts the pathway flux. MZ233 could also form from α-ionylideneacetic acid during the extraction procedure. However, no reducing agents were used.

ABA was predominantly detected in the supernatant and was only detectable in cell pellet with the increased sensitivity of HPLC-HRMS. Based on the ABA detection limit of the HPLC–MS protocol used, we estimate that the intracellular ABA concentration is below 10 µg/gCDW. For most applications it is desirable to have the compound-of-interest in the extracellular milieu, since it simplifies downstream processes and since intracellular accumulation of metabolites can trigger cellular stress responses.

The strain SCIGS22a has been shown to be well suited for the production of sesquiterpenes and sesquiterpenoids by providing high amounts of FPP, changing the NADH-NADPH ratio in favour of NADPH, and separating growth and production phase by the dynamic regulation of *ERG9* [43, 50]. Compared to a strain that only overexpresses tHMG1, the additional modifications of SCIGS22 resulted in a 4-fold increase in yield of the plant sesquiterpene α-santalene [50]. Precursor and co-factor supply did not affect ABA production as seen by comparing the strains DABA1, TABA1 and SABA1 (Fig. 2b). In contrast to ABA, α-santalene is produced from FPP by a single enzyme which can be expressed from a high-copy plasmid. Multi-step heterologous pathways provide a greater challenge since the activities of the individual enzymes need to be carefully balanced to ensure high flux towards the product. Indeed, by increasing the activity of the CYPs BcABA1 and BcABA2 we were able to increase the ABA titre 4.1-fold (Fig. 6a). Adding an additional copy of *bcaba1* alone (strain S3S1) doubled ABA production, while adding copies of *bcaba2* (S3S2) and *bcaba3* (S3S3) only slightly increased ABA titres. However, the ABA:MZ233 ratio of S3S1 and S3S2 differed greatly (Fig. 6b). Screening for pathway intermediates and side-products was crucial to realize that BcABA2 activity is a major limitation for ABA production after increasing BcABA1 activity. MZ233 is approximately 1/3 as abundant as ABA in S3S12, the strain reaching the highest ABA titres, whereas in S3S2 MZ233 is only 1/7 as abundant as ABA (Fig. 6b). This suggests that increasing the expression level of *bcaba2* would further channel the flux from MZ233 towards ABA.

CPRs act as co-enzymes for CYPs by facilitating the electron transfer from NADPH. When a heterologous CYP is expressed it is common practice to co-express the CPR from the original host. As expected, CPR activity is important for ABA production since the pathway includes two CYPs (Fig. 3b). In this case, even the *S. cerevisiae* CPR Ncp1 seems to be able to act as a redox partner for the *B. cinerea* enzymes. However, the lower OD_600_ for TABA5 suggest that high expression levels of Ncp1 are toxic. Cytotoxic effects of CPRs have been observed before and likely originate from formation of reactive oxygen species [61]. It is unknown why *NCP1* overexpression causes toxicity while *bccpr1* does not. A possible explanation could be that in contrast to BcCPR1, Ncp1 overexpression might change the activity of native CYPs, causing a metabolic imbalance. Concerning the importance of the *B. cinerea* CYPs in the ABA pathway, we assume that adjusting CPR expression levels will be required for increasing productivity. Strategies for optimising CYP-expressing yeast strains are described in the literature and these strategies might also be applicable for an ABA cell factory [62, 63].

The finding that ABA production mainly occurs during early growth was unexpected (Fig. 4). P_*PGK1*_ and P_*TEF1*_, used for the expression of all *B. cinerea* genes, are usually described as strong, constitutive promoters and are widely used in *S. cerevisiae* research [64]. A recent study investigated their expression profiles in batch culture and both promoters show a large difference in activity when comparing glucose and ethanol phase [65]. Especially for P_*PGK1*_ a strong decrease in activity was observed after the diauxic shift. Difference in gene expression levels would explain the slow increase of ABA titre after 16 h. However, in another report this growth phase dependant difference in expression level was less pronounced [64] and half-life of the expressed protein might play an important role in this regard. Since titres were increased several folds by increasing BcABA1 and BcABA2 abundance, it is unlikely that drainage of co-factors (e.g. heme required by CYPs) impedes ABA production after 16 h. For the same reason, product inhibition does not explain our observations. Further experiments are necessary to confirm that the reduced ABA production after 16 h is in fact due to the choice of promoters. In any case, for an efficient cell factory, promoters for expressing the individual ABA pathway genes will need to be chosen carefully depending on the fermentation conditions (e.g. batch or fed-batch cultivation).

Since a lot of effort has already been undergone to optimise the native metabolism for sesquiterpene and sesquiterpenoid production in *S. cerevisiae* [66, 67], we expect that increasing and balancing the activity of the ABA pathway enzymes will be the most important engineering approach for a high-yield ABA cell factory. Studies illustrate that heterologous enzymes with low catalytic efficiency can be exchanged for natural-occurring or mutagenized variants, by screening enzyme libraries. For example, by using a combination of enzymes from different organism the yield of the carotenoid lycopene was increased by 7.5-fold in *S. cerevisiae* [68]. Other ABA producing fungi like *Cercospora cruenta* could provide the material for a genetic library [69].

Other bottlenecks in improved strains could be oxygen supply and pH control. Labelling experiments from Inomata et al. [17] showed that all oxygen atoms in ABA originate from molecular oxygen, suggesting that aeration in bioreactors will be an important parameter. Especially when produced intracellularly, high titres of weak organic acids like ABA have been shown to cause severe cell stress, including high turgor pressure, inhibition of enzymes and increase in reactive oxygen species (ROS) [70, 71]. Even though low ABA titres did not have any negative effects on cell growth (Figures S5, S6 in Additional file 1), pH regulation or engineering the cellular response to acid stress might be necessary for a high-yield ABA cell factory. Alternatively, by adding a biocompatible organic layer (e.g. *n*-dodecane or isopropyl myristate oil) to the medium, potentially toxic products can be separated from the culture. Addition of isopropyl myristate oil proved beneficial for artemisinic acid production [46] and can furthermore simplify the extraction process. Yet it remains to be seen how the organic layer affects oxygen supply and the activity of the essential *B. cinerea* CYPs.

At the moment, *B. cinerea* is used for the biotechnological production of ABA, reaching titres higher than 1 g/L [72]. These concentrations were reached after about 9 days of cultivation in fed-batch culture with complex medium. For now, our ABA producing strains cannot compete with these titres. Our best producing-strain, S3S12, reached a titre of 11 mg/L (42 µM) with a carbon yield of 0.558 mg-ABA/g-glucose and 0.06 mg-ABA/g-glucose/OD_600_. Our proof-of-concept strain reaches less than 0.2% of what is theoretically possible based on a maximum yield of 0.325 g-ABA/g-glucose. In their study, Takino et al. [18] focused on elucidating the ABA pathway from *B. cinerea* in *A. oryzae*. They report ABA titres of 8 mg/L in an *A. oryzae* strain with plasmid-based expression of ABA pathway genes cultivated in complex medium with 3% maltose. The filamentous fungus could also be explored as a production host for ABA. However, studies confirm the potential of *S. cerevisiae* as high-yield cell factories for sesquiterpenes and sesquiterpenoids [45, 46], whereas we are not aware of comparable *A. oryzae* cell factories. One reason for the academic and industrial success story of *S. cerevisiae* is the large amount of knowledge and tools available, e.g. high-throughput screenings, CRISPR-based integration and gene regulation, and metabolic models [37–40]. From a sustainability and economic stand point it is essential to use affordable and abundant carbon sources as feed-stock. *S. cerevisiae* has simple nutrient requirements and can be engineered to use alternative carbon sources like xylose, which can be obtained from agricultural waste [73–75]. High-cell density bioprocesses are established, industrially robust and allow for scalable production. Considering these advantages, we believe that with continuing engineering efforts *S. cerevisiae* could become the preferred organism for ABA production.

## Conclusion

This study provides an example for sesquiterpenoid production in *S. cerevisiae* via a multi-step heterologous pathway. Our proof-of-concept strains utilise a 4-step metabolic pathway originating from *B. cinerea* to produce ABA. We were able to identify production bottlenecks, leading to more than 4-fold increased ABA titres in an improved strain, while by-product formation was reduced. The conducted experiments revealed promising engineering targets for future strain improvements, including further balancing of pathway gene expression levels to minimise MZ233 formation and increasing CYP activities to increase the pathway flux. Our findings regarding the importance of CPR overexpression for CYP-expressing yeast strains and the production dynamics in batch culture confirm previous studies and the results can be considered for the design of other cell factories. The main goal of this study was to investigate the plausibility of a yeast ABA cell factory for biotechnological applications that would allow for cost-competitive production in the long-term. However, heterologous expression of *B. cinerea* genes in *S. cerevisiae* can also provide valuable insight for understanding ABA biosynthesis and can help to further elucidate the pathway in the native host.

## Methods

### Microorganisms

**Fig. 7:**
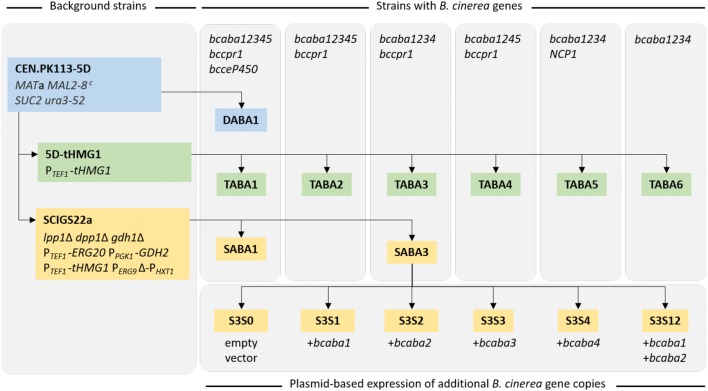
Pedigree chart of *S. cerevisiae* strains used in this study. The strains are colour-coded according to the background strain they originate from: blue = CEN.PK113-5D [76], green = 5D-tHMG1, yellow = SCIGS22a [43]. The integrated *B. cinerea* genes (or relevant *S. cerevisiae* genes in case of *NCP1*), regulated either by P_*TEF1*_ or P_*PGK1*_, are listed in the top part of the columns. The strains S3S1, S3S2, S3S3, S3S4 and S3S12 carry an additional copy of one or two *B. cinerea* genes expressed from the plasmid p416 GDP (*URA3*) (Table 3). More detailed information about the strains’ genotypes can be found in Table 1

*NEB*^*®*^ *5*-*alpha Competent E. coli* cells (New England Biolabs) were used for plasmid amplification. The *S. cerevisiae* strains used in this study are displayed as a strain pedigree chart in Fig. 7 and listed in detail in Table 1.

### Chemicals and media

LB medium was prepared with 10 g/L tryptone, 5 g/L yeast extract, 5 g/L NaCl, 5 mL/L 1 M Tris–HCl and 100 mg/L ampicillin. For agar plates, 20 g/L agar were added.

YPD medium was prepared with 10 g/L yeast extract, 20 g/L peptone from casein and 20 g/L glucose. For agar plates, 20 g/L agar were added. For plates with antibiotics, nourseothricin (clonNAT, 100 mg/L) and/or geneticin (G418, 200 mg/L) were added to the media.

Minimal medium (adapted from [77]) was prepared with 7.5 g/L (NH_4_)_2_SO_4_, 14.4 g/L KH_2_PO_4_, 0.5 g/L MgSO_4_·7H_2_O, 20 g/L glucose, 50 µg/L d-biotin, 1 mg/L d-pantothenic acid hemicalcium salt, 1 mg/L thiamin-HCl, 1 mg/L pyridoxin-HCl, 1 mg/L nicotinic acid, 0.2 mg/L 4-aminobenzoic acid, 25 mg/L myo-inositol, 6 mg/L FeSO_4_·7H_2_O, 9 mg/L ZnSO_4_·7H_2_O, 9 mg/L CaCl_2_·2H_2_O, 2 mg/L MnCl_2_·4H_2_O, 600 µg/L CoCl_2_·6H_2_O, 600 µg/L CuSO_4_·5H_2_O, 800 µg/L Na_2_MoO_4_·2H_2_O, 2 mg/L H_3_BO_3_, 200 µg/L KI, 38 mg/L Na_2_EDTA·2H_2_O. The pH was adjusted to 6.5. The medium was supplemented with 60 mg/L uracil for strains without plasmids.

The (*S*)-(+)-ABA standard was purchased from Cayman Chemicals (article number 10,073, purity ≥ 98%).

### Plasmid and strain construction

The sequences of the genes *ATCC*-*Bcin08g03850* (*bcaba1*), *ATCC*-*Bcin08g03840* (*bcaba2*), *ATCC*-*Bcin08g03880* (*bcaba3*), *ATCC*-*Bcin08g03830* (*bcaba4*), *ATCC*-*Bcin01g03520* (*bcaba5*), *ATCC*-*Bcin01g03510* (CYP gene; named *bcceP450* [21]) and *ATCC*-*Bcin12g03180* (NADPH cytochrome P450 reductase; named *bccpr1*) from the ABA overproducing *B. cinerea* strain ATCC58025 were ordered from Genscript (https://www.genscript.com), codon optimised for *S. cerevisiae* (see Additional file 3 for gene sequences). All sequencing was performed by Eurofins Genomics (https://www.eurofinsgenomics.eu).

Phusion High-Fidelity DNA polymerase (Thermo Fisher Scientific) was used for PCR of DNA fragments up to 3 kb, PrimeSTAR HS DNA Polymerase (Clontech) was used for PCRs with products longer than 3 kb. PCR products were digested with FastDigest DpnI (Thermo Fisher Scientific) for 2 h at 37 °C, before being purified using the GeneJET PCR Purification Kit (Thermo Fisher Scientific). For colony PCR, DreamTaq DNA polymerase (Thermo Fisher Scientific) was used (protocol according to Easyclone-MarkerFree manual, [37]).

The truncated gene corresponding to the catalytic domain of the protein Hmg1 from *S. cerevisiae* (YML075C) was integrated into the genome of CEN.PK113-5D using the vector pIRP01 as described previously [43].

For all other genomic integrations, the Easyclone-MarkerFree vector toolkit was used [37]. All constructed plasmids with information about integration sites and verification primers can be found in Table 2. The integration cassettes contained either one or two genes regulated by the strong promoters P_*PGK1*_ or P_*TEF1*_ [64]. The bidirectional p*PGK1*-p*TEF1* promoter brick was amplified from the plasmid pSP-GM2 [78]. *S. cerevisiae* transformations were based on the protocol by Gietz and Woods [79]. Instead of using USER enzyme cloning as stated in the Easyclone-MarkerFree manual [37], the plasmids were constructed with Gibson assembly (NEB, Gibson assembly Master Mix) following the instruction manual provided by the manufacturer. All primers used for plasmid construction are listed in Additional file 3. Plasmids were amplified in *E. coli* cultivated at 37 °C in 5 mL LB medium with ampicillin shaking at 200 rpm. The GeneJET Plasmid Miniprep Kit (ThermoFisher Scientific) was used for plasmid extraction from *E. coli*.

**Table 2:** Plasmids constructed for genomic integrations

Name	Plasmid backbone^a^	Integrated expression cassettes	Integration site (gRNA vector used)	Verification primers^a^
pCfB2904-ABA1	pCfB2904	P_*PGK1*_-*bcaba1*-T_*ADH1*_	XI-3 (pCfB3052)	2221/911
pCfB2904-ABA1-CPR	pCfB2904	P_*PGK1*_-*bcaba1*-T_*ADH1*_ P_*TEF1*_-*bccpr1*-T_*CYC1*_	XI-3 (pCfB3052)	2221/911
pCfB2904-ABA1-NCP1	pCfB2904	P_*PGK1*_-*bcaba1*-T_*ADH1*_ P_*TEF1*_-*NCP1*-T_*CYC1*_	XI-3 (pCfB3052)	2221/911
pCfB2909-ABA2-ABA4	pCfB2909	P_*PGK1*_-*bcaba2*-T_*ADH1*_ P_*TEF1*_-*bcaba4*-T_*CYC1*_	XII-5 (pCfB3052)	2221/899
pCfB3036-bcceP450	pCfB3036	P_*PGK1*_-*bcceP450*-T_*ADH1*_	XI-1 (pCfB3043)	2221/907
pCfB3035-ABA3-ABA5	pCfB3035	P_*PGK1*_-*bcaba5*-T_*ADH1*_ P_*TEF1*_-*bcaba3*-T_*CYC1*_	X-4 (pCfB3052)	2221/905
pCfB3035-ABA3	pCfB3035	P_*TEF1*_-*bcaba3*-T_*CYC1*_	X-4 (pCfB3052)	2221/905
pCfB3035-ABA5	pCfB3035	P_*PGK1*_-*bcaba5*-T_*ADH1*_	X-4 (pCfB3052)	2221/905

^a^See Easyclone-MarkerFree manual [37] for detailed sequence information

Gibson assembly was used to construct the plasmids for overexpression of ABA genes (Table 3, Additional file 3).

**Table 3:** Plasmids for overexpression of *B. cinerea* genes

Name	Plasmid backbone	Gene(s)	Reference
p416 (empty)	p416 GPD (*URA3*)	–	[80]
p416-ABA1	p416 GPD (*URA3*)	P_*TDH3*_-*bcaba1*	This study
p416-ABA2	p416 GPD (*URA3*)	P_*TDH3*_-*bcaba2*	This study
p416-ABA3	p416 GPD (*URA3*)	P_*TDH3*_-*bcaba3*	This study
p416-ABA4	p416 GPD (*URA3*)	P_*TDH3*_-*bcaba4*	This study
p416-ABA1+2	p416 GPD (*URA3*)	P_*TDH3*_-*bcaba1* P_*TEF1*_-*bcaba2*	This study

### *S. cerevisiae* cultivation

For the comparison of different background strains and investigating the effect of *bcaba3*, *bcaba5* and *bcceP450*, *S. cerevisiae* cultures were grown at 30 °C, while shaking at 200 rpm. A 5 mL preculture (minimal medium, in 50 mL falcon tubes) was inoculated by picking one yeast colony from a YPD plate. Three biological replicates were prepared for each strain. After approximately 24 h of cultivation, main cultures (20 mL minimal medium supplemented with uracil in 100 mL unbaffled shake flasks) were inoculated at an OD_600_ of 0.1 and grown for 48 h. After measuring the OD_600_, the cells were harvested by centrifugation (Sigma 4K15, 1500×*g*, 3 min) and the supernatant and cell pellet were separated for ABA extraction (see below). The pH of the supernatant was measured (Mettler Toledo SevenCompact pH Meter) and the cell pellet was washed with 20 mL distilled water and then lyophilized (Christ Alpha2-4 LSCbasic, 48 h, 1.030 mbar).

To monitor ABA production over time, the main culture volume was increased to 200 mL (minimal medium supplemented with uracil in 1 L unbaffled shake flasks). The OD_600_ was measured regularly and 15 mL culture samples were taken after 10 h, 16 h, 24 h, 36 h, 48 h and 58 h. The samples were then prepared for extraction as stated above.

The cultivation procedure was scaled-down for the experiments regarding the effects of CYP reductase overexpression and the plasmid-based ABA gene overexpression. The strains were cultivated in 24-square deep-well plates (Enzyscreen CR1224) with a culture volume of 2.5 mL. The plates were inoculated at an OD_600_ of 0.1 and incubated at 30 °C, while shaking at 250 rpm. Cells were harvested as described above.

### Extraction of ABA

Lyophilized biomass (20 mg) was transferred to a glass tube (Pyrex^®^ VISTA™ culture tube, screw-cap) and 4 mL methanol and 0.5 mL of glass beads (Merck, 0.5 mm) were added. For spiked samples, 40 µL of ABA standard (0.5 mg/mL in ethanol) were added to the 4 mL of methanol, resulting in an ABA concentration of 5 mg/L in the control samples. The tubes were vortexed (VWR^®^ Analog Multi-Tube Vortexer) for 30 min, centrifuged (Eppendorf Centrifuge 5804 R, 15 min, 3000×*g*, 4 °C) and 3 mL of supernatant were transferred to a new glass tube. The supernatant was evaporated completely (Genevac miVac, 45 °C, 2 h, 20 mBar) and reconstituted in 1 mL methanol. The tubes were vortexed for 30 min, centrifuged (15 min, 3000×*g*, 4 °C) and approximately 600 µL of supernatant were transferred to a HPLC vial.

Ethyl acetate with 0.5% (v/v) formic acid was used to extract the supernatant, since it has previously been shown to be suitable for ABA extraction from *B. cinerea* [20]. For each strain, 4 mL of supernatant were transferred to a glass tube and 4 mL EtOAc + 0.5% (v/v) formic acid were added. For spiked samples, 40 µL of ABA standard (0.5 mg/mL in ethanol) were added to the 4 mL culture before adding the extractant. The tubes were vortexed for 30 min and centrifuged (Eppendorf Centrifuge 5804 R, 15 min, 3000×*g*, 4 °C), before 3 mL of the upper layer were transferred to a new tube and evaporated (Genevac miVac, 45 °C, 2 h, 20 mBar). The residues were re-dissolved in methanol as described above and transferred to HPLC vials.

For the experiments conducted in 24-square deep-well plates, the extraction procedure was simplified and scaled-down. 1 mL of supernatant was freeze-dried in 1.5 mL Eppendorf tubes for 48 h. 1 mL of methanol was added, the samples were vortexed for 30 min (VWR^®^ Analog Multi-Tube Vortexer) and centrifuged for 15 min (Eppendorf Centrifuge 5417 R, 15 min, 3000×*g*, 4 °C). The supernatant was transferred to HPLC vials.

All extracted samples were stored at − 20 °C until the analysis.

### Detection of ABA by HPLC–MS

Presence of ABA in the supernatant and dried cell pellets was monitored using high-performance liquid chromatography coupled to mass spectrometry (HPLC–MS). Monitoring of the desired low-resolution mass was performed using an Agilent 6120 Single Quadrupole mass spectrometer coupled to an Agilent Infinity 1260 HPLC system consisting of a binary pump, autosampler, thermostat column compartment, and diode array detector (DAD). The mass spectrometer was operated with atmospheric pressure electrospray ionization (API-ES) source in both positive and negative mode, and the DAD monitored at 254 nm. Metabolites were analysed on an Agilent Poroshell 120 EC-C18 (2.7 μm, 3.0 × 50 mm) column (maintained at 40 °C) with a water-acetonitrile (MeCN) gradient solvent system containing 0.1% acetic acid. The LC gradient started at 95% water with acetic acid and ramped to 95% MeCN with acetic acid over 5 min, and held there for 2 min. Flow was set at 0.4 mL/min. The sample injection volume was set to 1 µL for samples originating from shake flasks cultures, while 8 µL were injected for samples from 24-square deep-well plates. HPLC–MS data were analysed using the MassHunter Qualitative Analysis Navigator (Agilent Technologies).

For quantification of ABA, an extracted ion chromatogram (EIC) for *m/z* 265 (ABA, M+H) was used. A calibration curve was prepared by diluting the (*S*)-(+)-ABA standard in MeOH to 36 mg/L, 18 mg/L, 9 mg/L, 4.5 mg/L and 2.25 mg/L. The Student's t-test (one-tailed, homoscedastic) was used to determine significance.

### HPLC-HRMS and metabolomics analysis

High-resolution mass spectrometry (HRMS) analysis was performed on an Agilent 6520 quadrupole time-of-flight (qTOF) mass spectrometer and coupled to an Agilent Infinity 1290 HPLC instrument. Metabolites were analysed on an UPLC HSS T3 (1.8 μm, 2.1 × 100 mm, Waters) column with a water-MeOH gradient solvent system containing 0.04% formic acid. The gradient started at 5% MeOH with formic acid (MPB) and ramped to 100% MPB over 6 min and held for 4.50 min at 100% MPB. Column temperature was set to 45 °C and the flow at 0.4 mL/min. Mass spectra was acquired using a Dual ESI source in either positive or negative mode scanning from 50 to 1700 *m/z* at 1.67 spectra/second. The capillary voltage was set at 3500 V. The source parameters were set with a gas temperature at 175 °C, gas flow at 10 L/min, and nebulizer at 45 psig. MS data was acquired with Mass Hunter Workstation Data Acquisition (Agilent Technologies).

Prior to analysis, each sample extract was dissolved in 1 mL methanol. During data acquisition, a methanol blank was run prior to the first sample injection and a methanol wash was performed after each sample set. QC sample was prepared from 10 random samples and used to confirm system suitability. The centroid MS data were processed with MassHunter Profinder (Agilent Technologies) and used to extract possible molecular features (MOFs) using the recursive untargeted feature-finding algorithm. The extracted MOFs were used for further downstream processing using Mass Profiler Professional (MPP, Agilent Technologies), where the extracts from the background samples were compared to their respective extract from the ABA-containing strains. A unique list of ions was selected using significant analysis, where MOFs were filtered based on their p-value (T-test unpaired, multiple testing correction using Benjamini-Hochberg, p-value < 0.001). A fold-change cut off of 8 was selected for the supernatant and 2 for the cell pellet.

Tandem MS/MS analysis was performed using a targeted auto-MS2 mode selecting for the MOFs present in the preferred unique ion list acquired for each sample. MS2 data were acquired in the positive mode from 100 to 1700 *m/z* with a MS scan rate of 4 spectra/s and a MS/MS scan rate of 2 spectra/s. Masses were fragmented with 10, 20, and 40 collision energy. The same LC method and column was used as described above.

### Growth profiler

A Growth Profiler 960 (Enzyscreen) was used to determine the growth profiles of different *S. cerevisiae* strains. Precultures were prepared as mentioned before. 250 µL cultures (minimal medium supplemented with uracil) were inoculated at an OD of 0.1 in a 96-well plate (Enzyscreen CR1496d).

## Supplementary information

**Additional file 1.** Additional analysis (HPLC–MS chromatograms, MS spectra, growth profiles). HPLC–MS chromatograms for 5D, 5D-tHMG1, SCIGS22a, DABA1, TABA1, TABA2, TABA3, TABA4, SABA1, 5D-tHMG1 spiked with ABA standard and a blank methanol run are shown. In addition, the file contains MS spectra for 5D-tHMG1, TABA1 and ABA standard. Growth profiles for the above-mentioned background and engineered strains are also displayed.

**Additional file 2.** Detected molecular features in HPLC-HRMS analysis. Observed molecular features with m/z between 200 and 270 detected with HPLC-HRMS in cell pellet and supernatant of TABA3 after 24 h. All molecular features detected in the background strain 5D-tHMG1 without B. cinerea genes have been excluded. Molecular formulae (MF) were postulated for the most abundant compounds (ion count higher than 106) and the ppm error calculated.

**Additional file 3.** Gene and primer sequences. File contains *B. cinerea* gene sequences codon-optimised for *S. cerevisiae* and list of PCR primers used for the construction of integration and expression vectors by Gibson assembly.

## Data Availability

The datasets used and analysed during the current study are available from the corresponding author on reasonable request.
