# Supplementary material for: Integration of a multi-step heterologous pathway in Saccharomyces cerevisiae for the production of abscisic acid
Source: Microb Cell Fact. 2019 Nov 25;18:205. doi: 10.1186/s12934-019-1257-z (PMC6876084; doi:10.1186/s12934-019-1257-z)
Supplement: Supplementary file 1 — Additional file 1. Additional analysis (HPLC–MS chromatograms, MS spectra, growth profiles). HPLC–MS chromatograms for 5D, 5D-tHMG1, SCIGS22a, DABA1, TABA1, TABA2, TABA3, TABA4, SABA1, 5D-tHMG1 spiked with ABA standard and a blank methanol run are shown. In addition, the file contains MS spectra for 5D-tHMG1, TABA1 and ABA standard. Growth profiles for the above-mentioned background and engineered strains are also displayed. [file 12934_2019_1257_MOESM1_ESM.pdf]

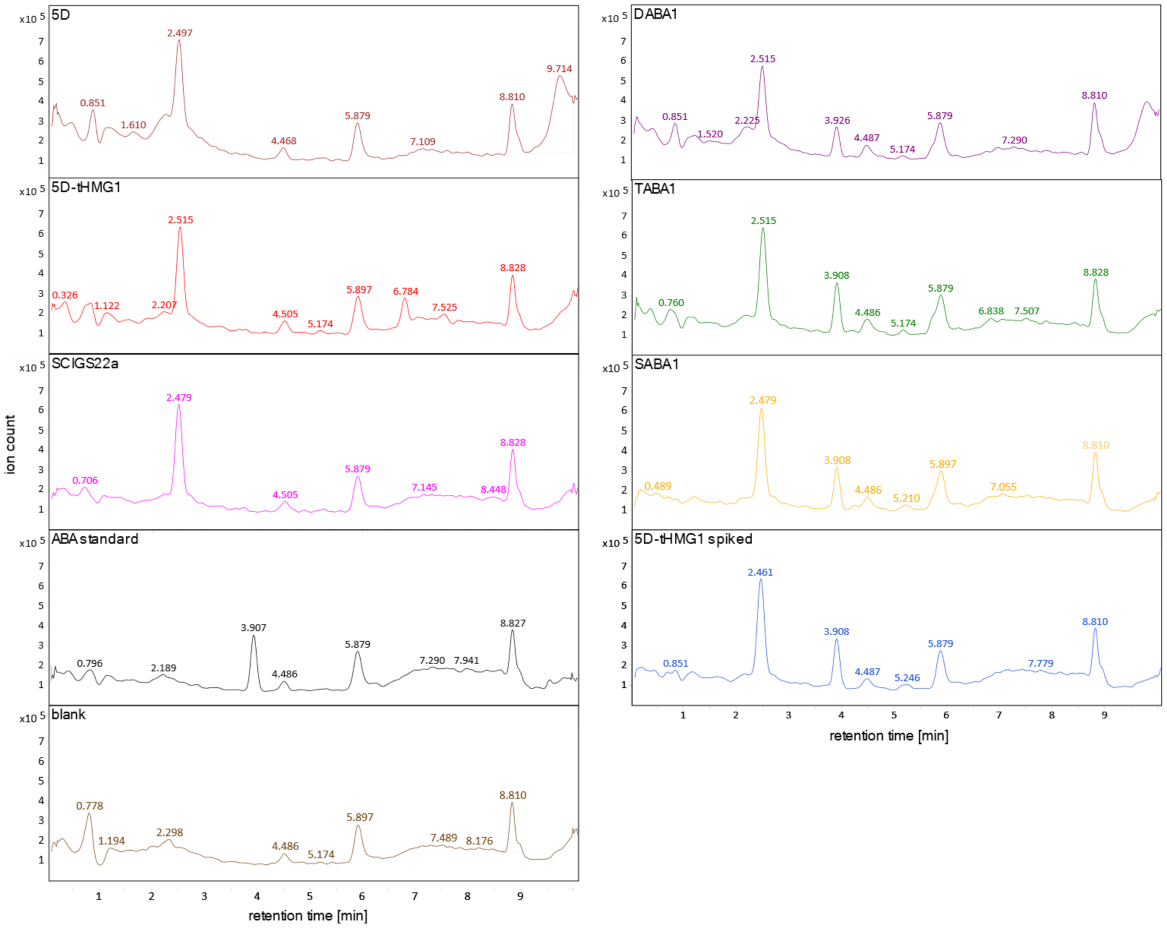

Figure S1: Chromatograms (smoothed) from HPLC-MS analysis. Shown are extracted supernatants after 48 h of cultivation ¬of the strains: 5D, 5D-tHMG1, SCIGS22a, DABA1, TABA1, SABA1, 5D-tHMG1 spiked with ABA standard, as well as (*S*)-(+)-ABA standard dissolved in MeOH and a blank run with MeOH. One replicate is displayed per strain. Retention time is displayed on top of the peaks.

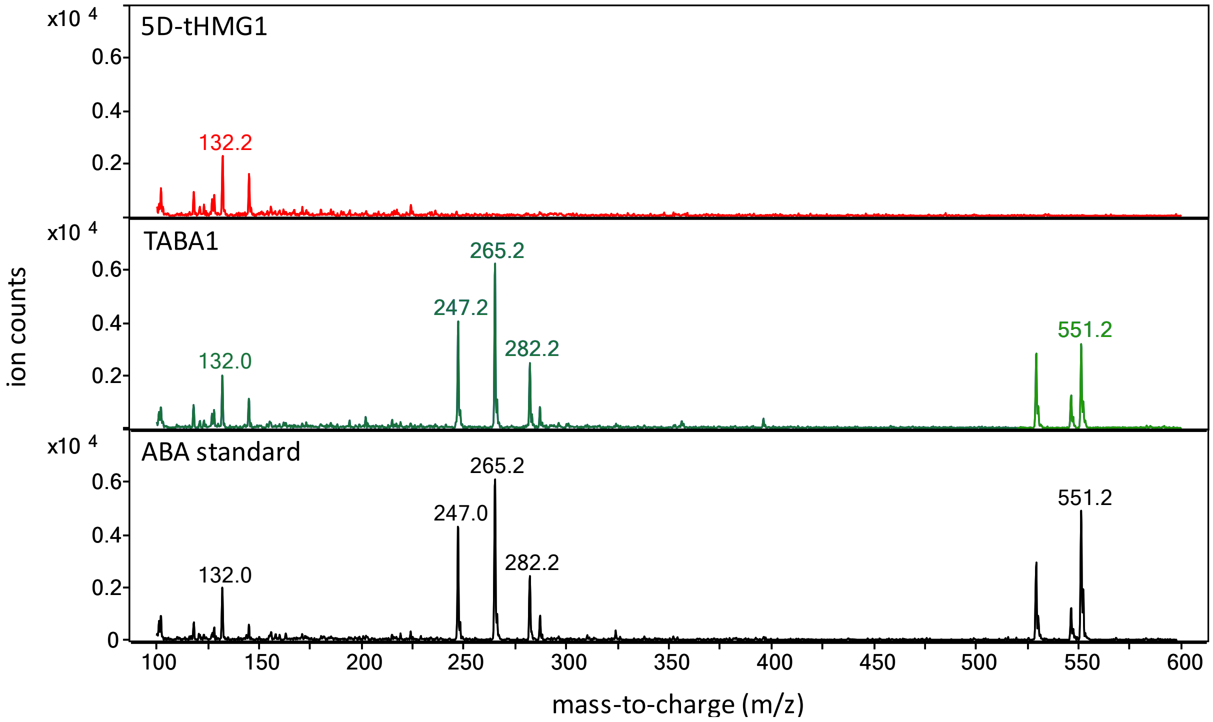

Figure S2: MS spectra of peaks at retention time 3.908 min. Spectra are derived from extracted supernatants after 48 h of cultivation of the strains 5D-tHMG1 (peak manually defined between 3.9 min and 4.0 min) and TABA1 as well as the (*S*)-(+)-ABA standard dissolved in MeOH. For DABA1 and ABA1 the same *m/z* peaks were observed as for TABA1 (data not shown). One replicate is displayed per strain. The *m/z* ratios are displayed above the peaks.

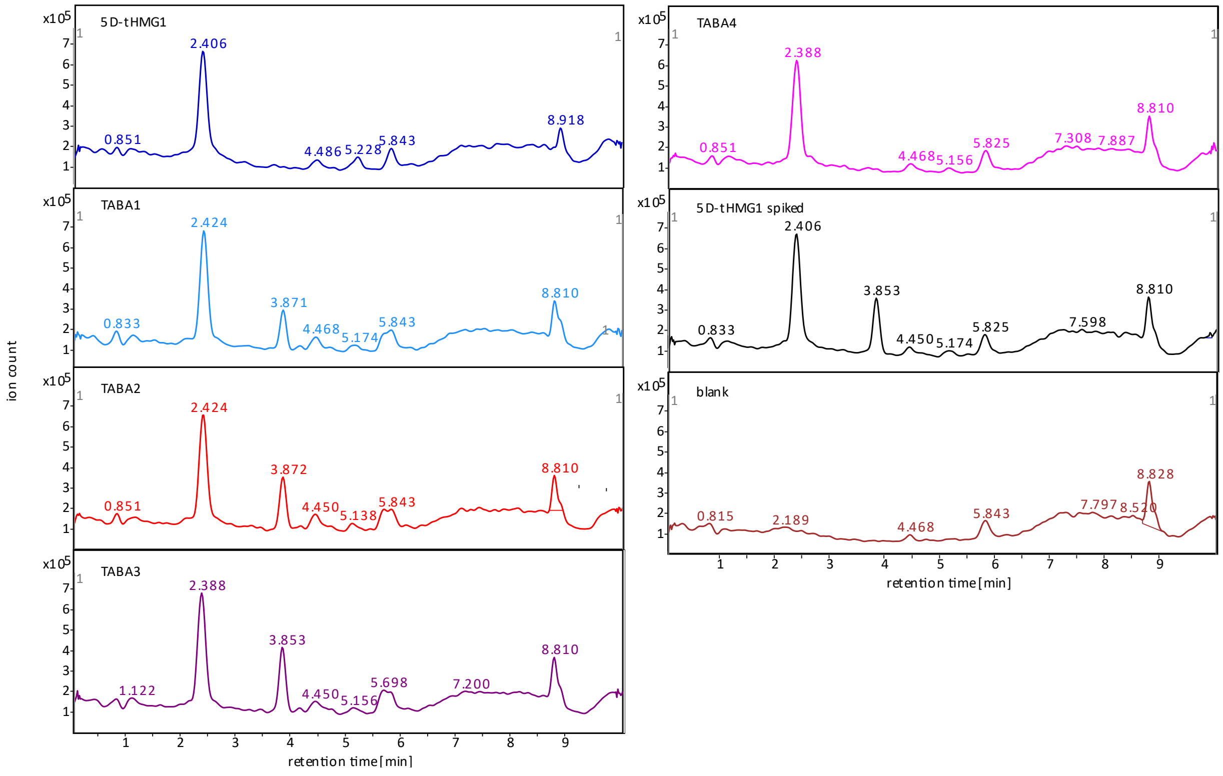

Figure S3: Chromatograms (smoothed) from HPLC-MS analysis. Shown are results from extracted supernatants after 48 h of cultivation of the strains: 5D-tHMG1, TABA1, TABA2, TABA3, TABA4 as well as 5D-tHMG1 spiked with ABA standard and a blank run with MeOH. One replicate is displayed per strain. Retention time is displayed on top of the peaks. ABA observed at 3.853 minutes.

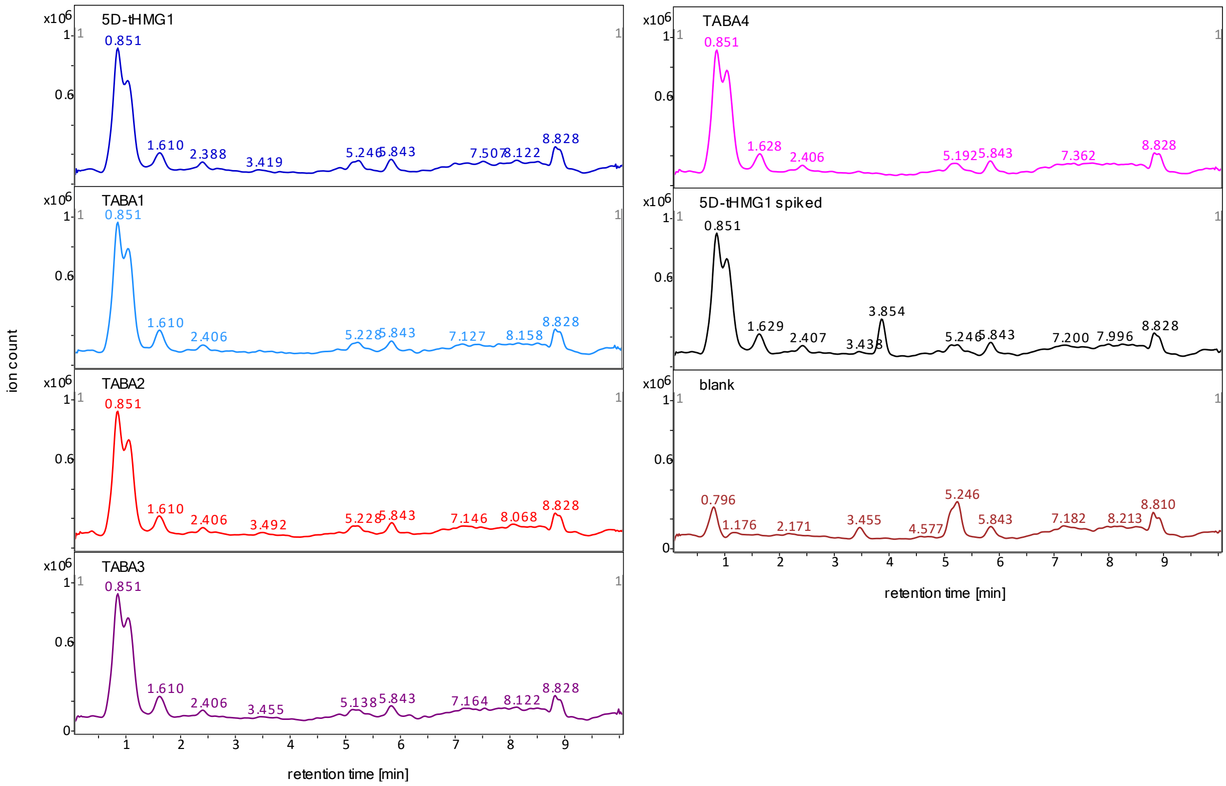

Figure S4: Chromatograms (smoothed) from HPLC-MS analysis. Shown are results from extracted pellets after 48 h of cultivation of the strains: 5D-tHMG1, TABA1, TABA2, TABA3, TABA4 as well as 5D-tHMG1 spiked with ABA standard and a blank run with MeOH. One replicate is displayed per strain. Retention time is displayed on top of the peaks.

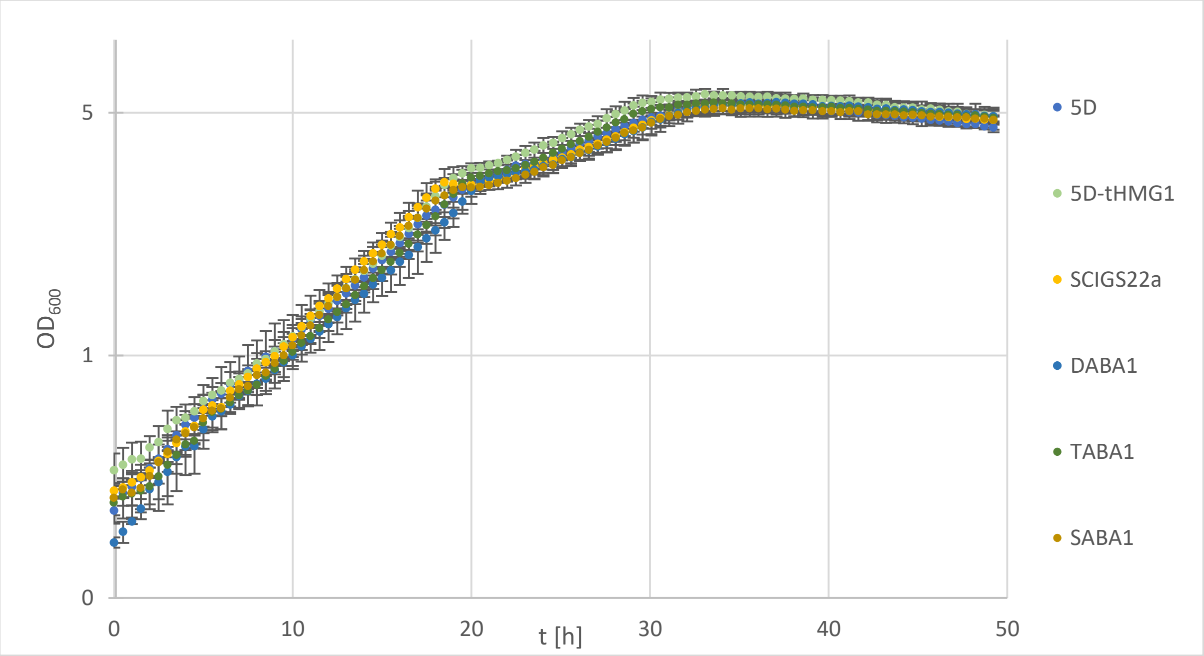

Figure S5: Growth profiles of different background and engineered strains containing *bcaba12345*, *bccpr1*, and *bcceP450*. A Growth Profiler 960 (Enzyscreen) was used to monitor the OD_{600} for 48 h, with measurements every 30 min. Cultures were inoculated at OD_{600} 0.1 in minimal medium supplemented with uracil and shaken at 250 rpm at 30 °C. Average and standard deviation of three independent biological replicates are shown. OD_{600} is displayed on a logarithmic scale.

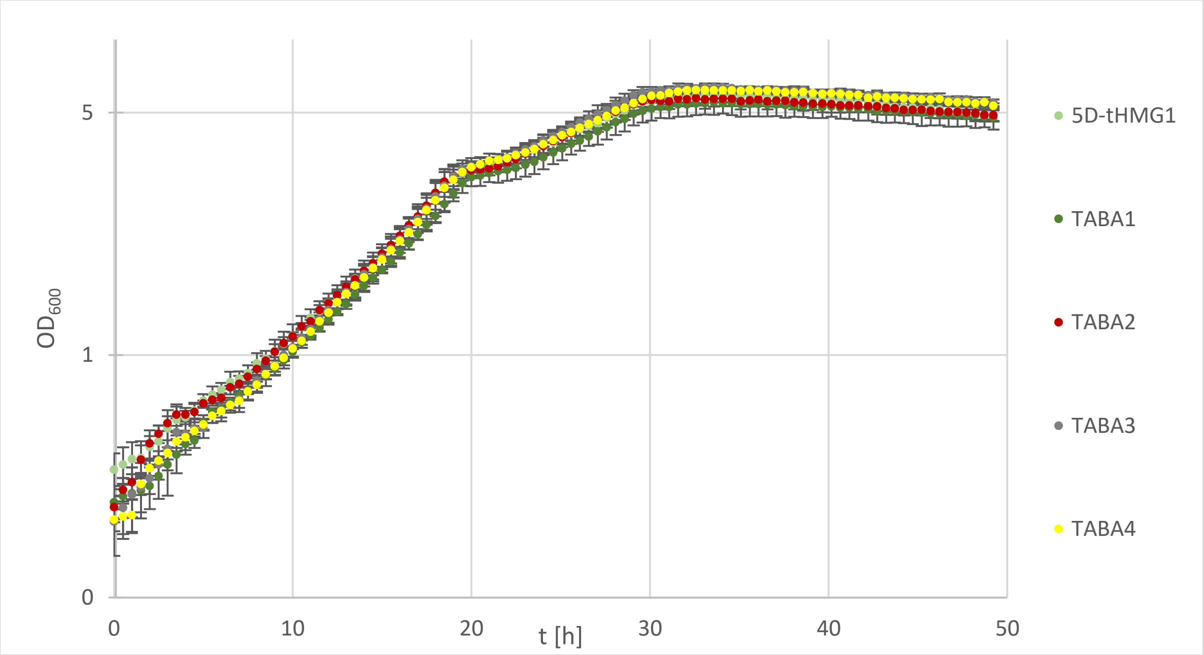

Figure S6: Growth profiles of strains based on the 5D-tHMG1 background strain. A Growth Profiler 960 (Enzyscreen) was used to monitor the OD_{600} for 48 h, with measurements taken every 30 min. Cultures were inoculated at OD_{600} 0.1 in minimal medium supplemented with uracil and shaken at 250 rpm at 30 °C. Average and standard deviation of three independent biological replicates are shown. OD_{600} is displayed on a logarithmic scale.
